# Supplementary material for: Host Cell Signatures of the Envelopment Site within Beta-Herpes Virions
Source: Int J Mol Sci. 2022 Sep 1;23(17):9994. doi: 10.3390/ijms23179994 (PMC9456339; doi:10.3390/ijms23179994)
Supplement: Supplementary file 1 [file ijms-23-09994-s001.zip › ijms-1850137-supplementary.pdf]

Table S1. Tethering complexes in the membrane system

Location	Tethering complex/protein	Trafficking route	Components */**	Reference
PM	Exocyst	ERC → PM	EXOC1, EXOC2, EXOC3*, EXOC4 and EXOC5, EXOC6, EXOC7, EXOC8	[14,15]
	Tomosyn/Sro7	RE → PM	STXBP1 + ???	
	Myosin V			
EE	CORVET	EE → EE; EE → LE	VPS11 – VPS16 – VPS18 – VPS3 – VPS8 – VPS33A/B	[16]
	Mini CORVET		VPS8, VPS18, VPS33A and VPS16A	
	EEA1	PM → EE; EE → EE	EEA1	
	FERARI	EE → ERC	VPS45, VIPAS39, rabenosyn5, EHD1/3**, ANK1-3, RAB11FIP5	[13]
RE	EARP	EE → RE	VPS51, VPS52, VPS53, VPS50 (Syndetin/VPS54L)	[16]
ERC	CHEVI		VIPAR (VIPAS39/VPS16B), VPS33B	
LE/Ly	HOPS	EE → LE → Ly	VPS11-VPS16-VPS18-VPS39- VPS41-VPS33A	[15,16]
TGN	Golgin 97	TGN → EE; EE → TGN ??		[15]
	Gcc-185	EE → TGN	GCC2	
	Golgin-245	TGN → EE; EE → TGN	GOLGA4	
	GARP	EE → TGN	VPS51-VPS52-VPS53-VPS54	
	Gcc88		GCC1	
	Giantin	ER→cGolgi	GOLGB1	
	CASP		CUX1	
	TRAP III		BET3A, BET3B, BET5, TRS20, TRS23, TRS31, TRS33, TRS85	
Golgi	TMF	Later Golgi	TMF1	
	COG	Intra Golgi	COG1-COG2-COG3-COG4 and COG5-COG6-COG7-COG8	[15]
	Golgin84	cGolgi → mGolgi	GOLGA5	
	TRAP II		BET3A, BET3B, BET5, TRS20, TRS23, TRS31, TRS33, TRS65, TRS120, TRS130	[15]
cisGolgi	CASP	Intra Golgi		[15]
	GMAP-210	ER → cGolgi; cGolgi → mGolgi	TRIP11	
	COG	Golgi → ER	COG1-COG2-COG3-COG 4 and COG5-COG6-COG7-COG 8	
	GM130	ER → cGolgi	GM130	
	p115	ER → cGolgi		
	TRAP I		BET3A, BET3B, BET5, TRS20, TRS23, TRS31, TRS33	
ER	Dsl1/NRZ	Golgi → ER	DSl1, TIP20, DSl3/SEC39	[15,17]

* Components identified in virion preparations are underlined.

** Components identified in proteinase K-treated virion preparations are underlined and labeled in red.

PM, plasma membrane; EE, early endosome; RE, recycling endosome; ERC, endosomal recycling compartment; TGN, trans-Golgi network; LE, late endosome; ER, endoplasmic reticulum

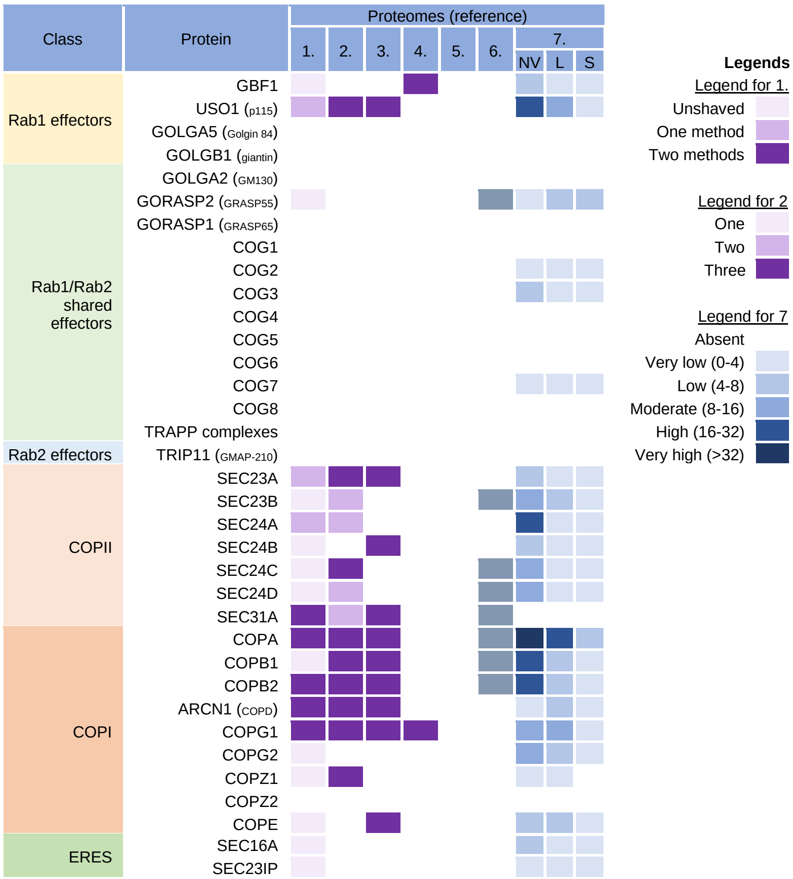

Figure S1. Identification of intermediate compartment (IC) proteins in virion and extracellular nanoparticle preparations. The proteins known to associate with membranes of the IC (based on references [1]), were identified in five proteomes of HCMV virion preparations (proteome 1-5), proteomes of HSV-1 heavy particles (proteome 6), and proteomes of non-vesicular extracellular particles (NVEP), large (L) and small (S) extracellular vesicles (proteome 7). Proteome 1 [2] presents data for untreated HCMV virion preparations (unshaved) and the same preparation treated with proteinase K ("shaved" virions). Identification of cargo proteins in shaved virion preparations is marked with different color codes depending on whether they were detected by one or two mass spectrometric methods. Proteome 2 [3] displays data in different color codes depending on the identification of a cargo protein in one, two, or three biological replicates. Proteomes 3-6 (proteomes 3 [4], 4 [5], 5 [6], and 6 [7]) indicate whether a protein is present or not. Proteome 7 [8] shows the abundance of a cargo protein in high-resolution density gradient-purified non-vesicular (NV), *l*EVs (L) and *s*EVs (S) samples of DKO-1 cells. The color code very low means less than 2xlog2 abundance relative to the average signal [8]. Low means 2-3x, moderate 3-4x, high 4-5x and very high ≥6x.

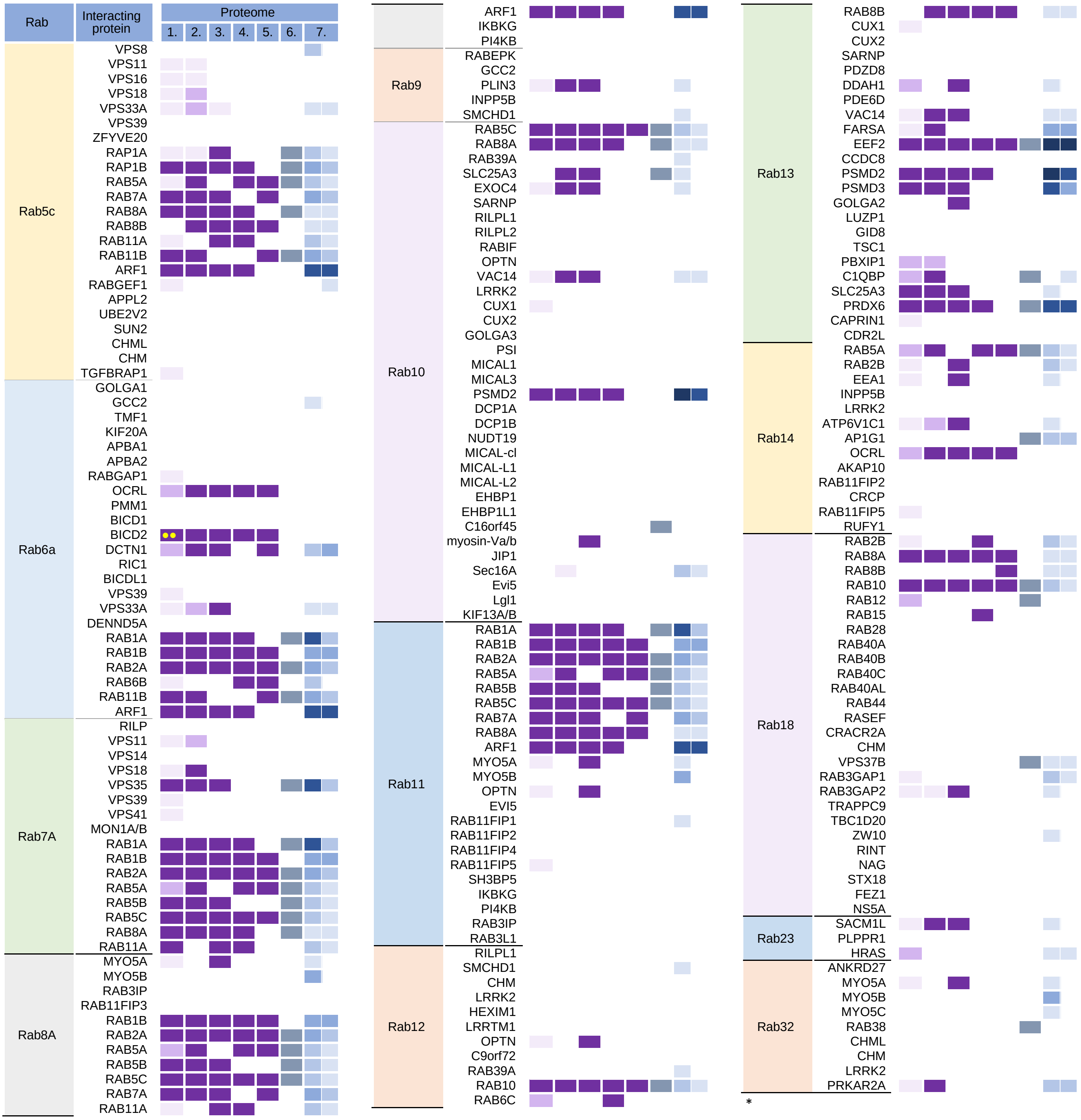

Figure S2. Identification of Rab interactors in virion and extracellular nanoparticle preparations. The experimentally determined proteins known to build these complexes (based on STRING Interaction Network; https://string-db.org/ accessed on June 5, 2022), were identified in five proteomes of HCMV virion preparations (proteome 1-5), proteomes of HSV-1 heavy particles (proteome 6), and proteomes of non-vesicular extracellular particles (NVEP) (proteome 7). Proteome 1 [2] presents data for untreated HCMV virion preparations (unshaved) and the same preparation treated with proteinase K ("shaved" virions). Identification of cargo proteins in shaved virion preparations is marked with different color codes depending on whether they were detected by one or two mass spectrometric methods. Proteome 2 [3] displays data in different color codes depending on the identification of a cargo protein in one, two, or three biological replicates. Proteomes 3-6 (proteomes 3 [4], 4 [5], 5 [6], and 6 [7]) indicate whether or not a protein is present. Proteome 7 [8] shows the abundance of a cargo protein in high-resolution density gradient-purified non-vesicular (NV) samples of DKO-1 cells (left box) and Gli36 cells (right box). The color code very low means less than 2xlog2 abundance relative to the average signal [8]. Low means 2-3x, moderate 3-4x, high 4-5x and very high ≥6x.

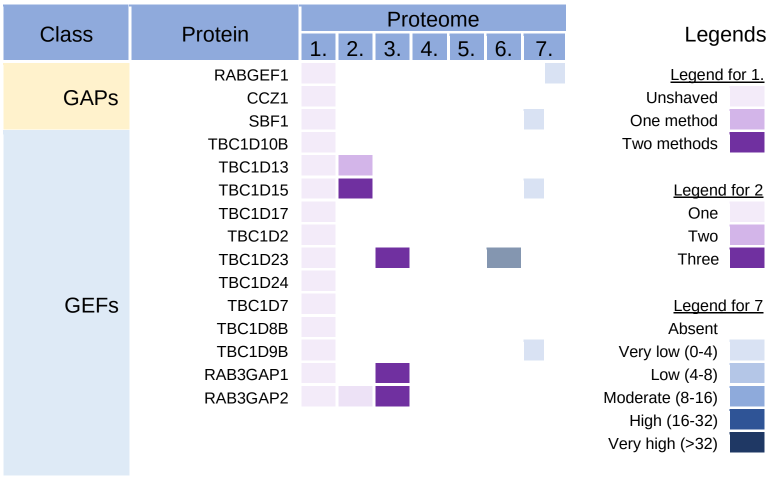

Figure S3. Identification of Rab GEFs and GAPs in virion preparations and extracellular nanoparticle preparations. The proteins known to build these complexes (based on reference [9]), were identified in five proteomes of HCMV virion preparations (proteome 1-5), proteomes of HSV-1 heavy particles (proteome 6), and non-vesicular extracellular particles (proteome 7). Proteome 1 [2] presents data for untreated HCMV virion preparations (unshaved) and the same preparation treated with proteinase K ("shaved" virions). Identification of cargo proteins in shaved virion preparations is marked with different color codes depending on whether they were detected by one or two mass spectrometric methods. Proteome 2 [3] displays data in different color codes depending on the identification of a cargo protein in one, two, or three biological replicates. Proteomes 3-6 (proteomes 3 [4], 4 [5], 5 [6], and 6 [7]) indicate whether or not a protein is present. Proteome 7 [8] shows the abundance of a cargo protein in high-resolution density gradient-purified non-vesicular (NV) samples of DKO-1 cells (left box) and Gli36 cells (right box). The color code very low means less than 2x log2 abundance relative to the average signal [8]. Low means 2-3x, moderate 3-4x, high 4-5x and very high ≥6x.

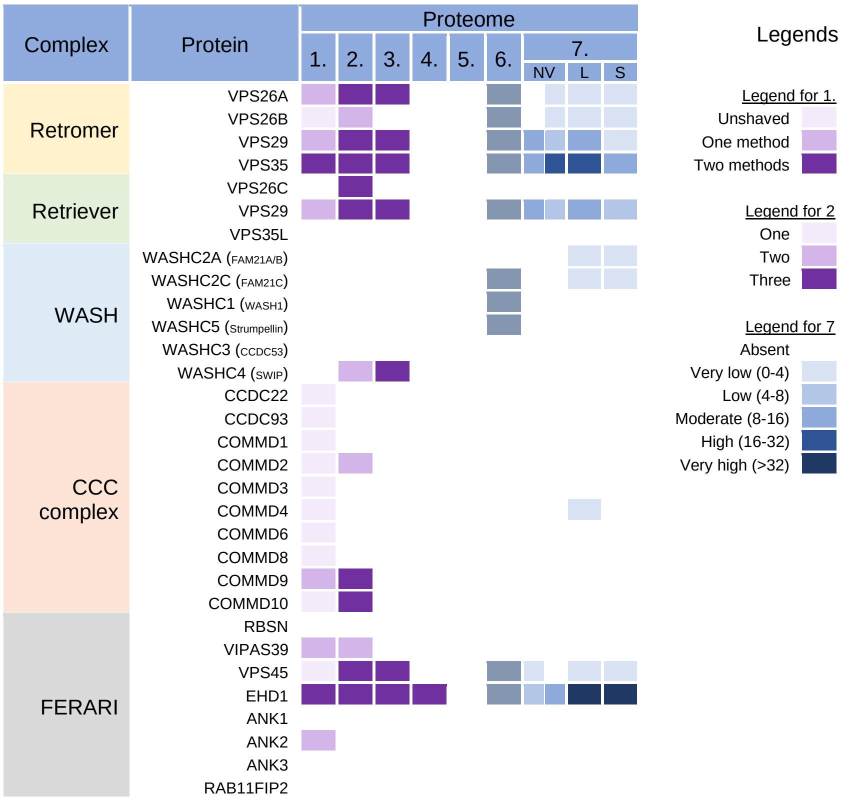

Figure S4. Identification of components of retrieving complexes (retromer, retriever, WASH, and CCC complexes) and FERARI complex in virion and extracellular nanoparticle preparations. The proteins known to build these complexes (based on references [10–13]), were identified in five proteomes of HCMV virion preparations (proteome 1-5), proteomes of HSV-1 heavy particles (proteome 6), and proteomes of non-vesicular extracellular particles (NVEP), large (L) and small (S) extracellular vesicles (proteome 7). Proteome 1 [2] presents data for untreated HCMV virion preparations (unshaved) and the same preparation treated with proteinase K ("shaved" virions). Identification of cargo proteins in shaved virion preparations is marked with different color codes depending on whether they were detected by one or two mass spectrometric methods. Proteome 2 [3] displays data in different color codes depending on the identification of a cargo protein in one, two, or three biological replicates. Proteomes 3-6 (proteomes 3 [4], 4 [5], 5 [6], and 6 [7]) indicate whether or not a protein is present. Proteome 7 [8] shows the abundance of a cargo protein in high-resolution density gradient-purified non-vesicular (NV) samples of DKO-1 cells (left box) or Gli36 cells (right box) and lEVs (L) and sEVs (S) samples of DKO-1 cells. The color code very low means less than 2x log2 abundance relative to the average signal [8]. Low means 2-3x, moderate 3-4x, high 4-5x and very high ≥6x.

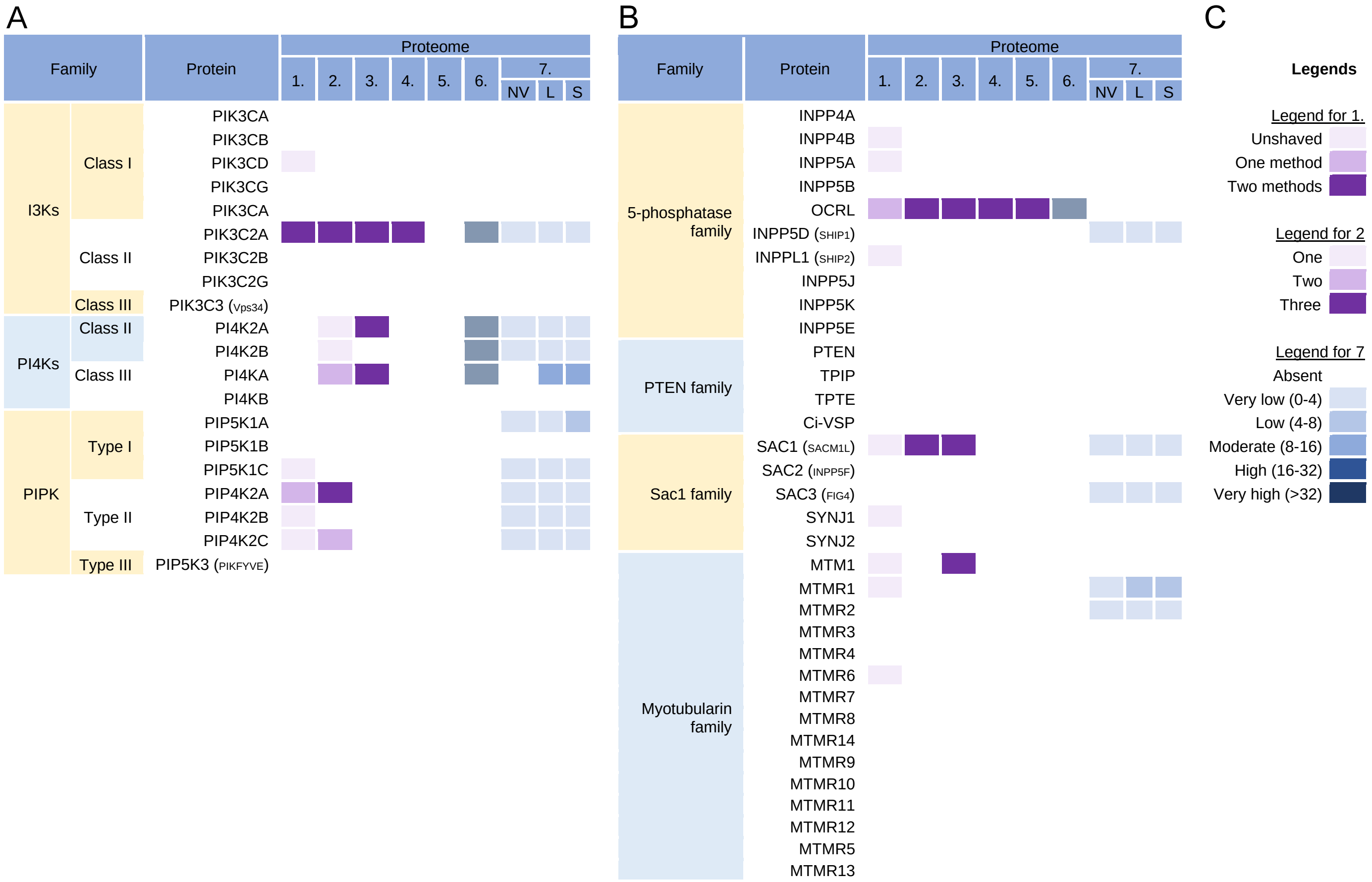

Figure S5. Identification of phosphoinositide kinases and phosphatases in virion and extracellular nanoparticle preparations. The kinases (**A**) and phosphatases (**B**) (based on references [18] and [19], respectively), were identified in five proteomes of HCMV virion preparations (proteome 1-5), proteomes of HSV-1 heavy particles (proteome 6), and proteomes of non-vesicular extracellular particles (NVEP), large (L) and small (S) extracellular vesicles (proteome 7). Proteome 1 [2] presents data for untreated HCMV virion preparations (unshaved) and the same preparation treated with proteinase K ("shaved" virions). Identification of cargo proteins in shaved virion preparations is marked with different color codes (**C**) depending on whether they were detected by one or two mass spectrometric methods. Proteome 2 [3] displays data in different color codes (**C**) depending on the identification of a cargo protein in one, two, or three biological replicates. Proteomes 3-6 (proteomes 3 [4], 4 [5], 5 [6], and 6 [7]) indicate whether or not a protein is present. Proteome 7 [8] shows the abundance of a cargo protein in high-resolution density gradient-purified non-vesicular (NV) samples of DKO-1 cells (left box) or Gli36 cells (right box) and lEVs (L) and sEVs (S) samples of DKO-1 cells. The color code (**C**) very low means less than 2x log2 abundance relative to the average signal [8]. Low means 2-3x, moderate 3-4x, high 4-5x and very high ≥6x.

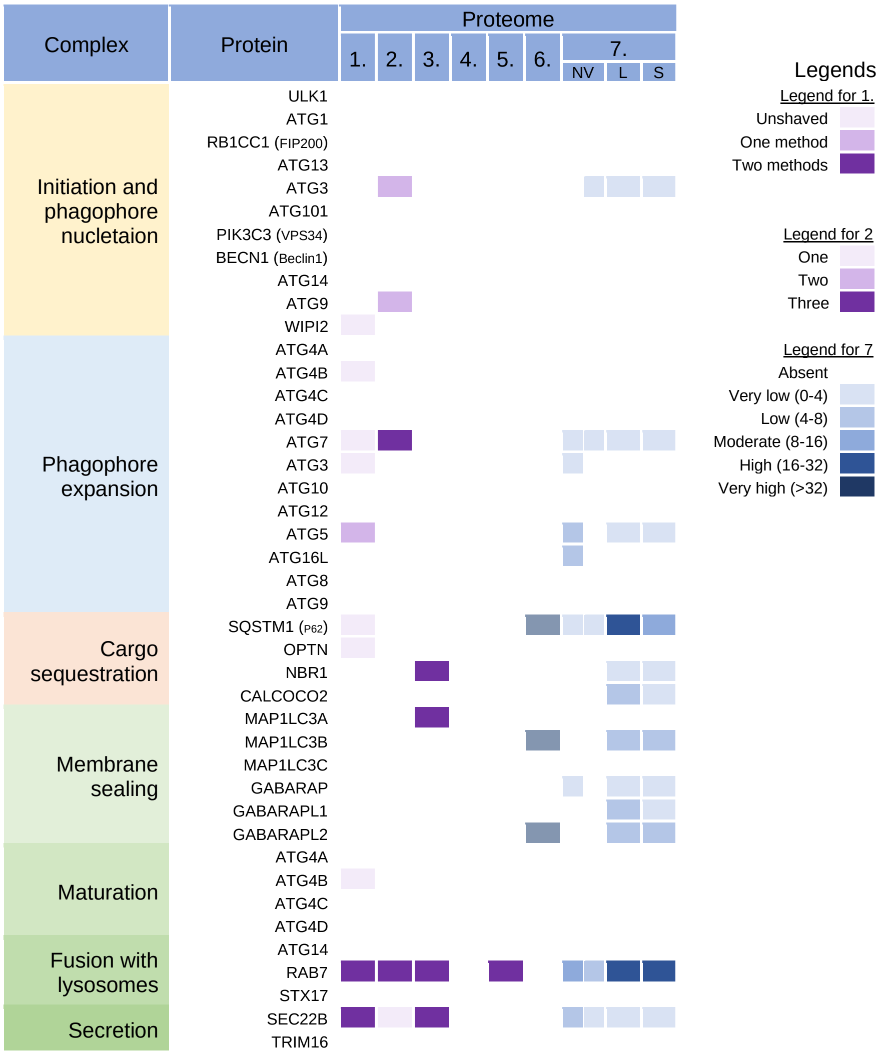

Figure S6. Identification of the key autophagic factors in virion and extracellular nanoparticle preparations. The proteins known to contribute to these processes (based on references [20–22]), were identified in five proteomes of HCMV virion preparations (proteome 1-5), proteomes of HSV-1 heavy particles (proteome 6), and proteomes of non-vesicular extracellular particles (NVEP), large (L) and small (S) extracellular vesicles (proteome 7). Proteome 1 [2] presents data for untreated HCMV virion preparations (unshaved) and the same preparation treated with proteinase K ("shaved" virions). Identification of cargo proteins in shaved virion preparations is marked with different color codes depending on whether they were detected by one or two mass spectrometric methods. Proteome 2 [3] displays data in different color codes depending on the identification of a cargo protein in one, two, or three biological replicates. Proteomes 3-6 (proteomes 3 [4], 4 [5], 5 [6], and 6 [7]) indicate whether or not a protein is present. Proteome 7 [8] shows the abundance of a cargo protein in high-resolution density gradient-purified non-vesicular (NV) samples of DKO-1 cells (left box) or Gli36 cells (right box) and lEVs (L) and sEVs (S) samples of DKO-1 cells. The color code very low means less than 2x log2 abundance relative to the average signal [8]. Low means 2-3x, moderate 3-4x, high 4-5x and very high ≥6x.

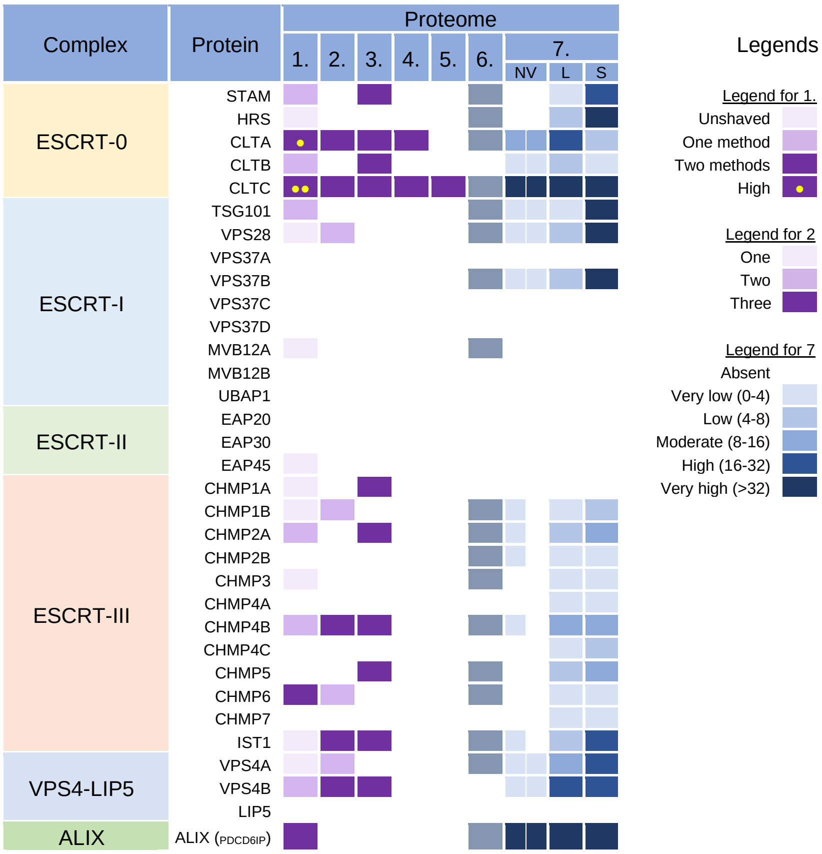

Figure S7. Identification of components of the ESCRT machinery in virion preparations and extracellular nanoparticle preparations. The proteins known to contribute to these processes (based on reference [23]), were identified in five proteomes of HCMV virion preparations (proteome 1-5), proteomes of HSV-1 heavy particles (proteome 6), and proteomes of non-vesicular extracellular particles (NVEP), large (L) and small (S) extracellular vesicles (proteome 7). Proteome 1 [2] presents data for untreated HCMV virion preparations (unshaved) and the same preparation treated with proteinase K ("shaved" virions). Identification of cargo proteins in shaved virion preparations is marked with different color codes depending on whether they were detected by one or two mass spectrometric methods. Proteome 2 [3] displays data in different color codes depending on the identification of a cargo protein in one, two, or three biological replicates. Proteomes 3-6 (proteomes 3 [4], 4 [5], 5 [6], and 6 [7]) indicate whether or not a protein is present. Proteome 7 [8] shows the abundance of a cargo protein in high-resolution density gradient-purified non-vesicular (NV) samples of DKO-1 cells (left box) or Gli36 cells (right box) and lEVs (L) and sEVs (S) samples of DKO-1 cells. The color code very low means less than 2x log2 abundance relative to the average signal [8]. Low means 2-3x, moderate 3-4x, high 4-5x and very high ≥6x.

Supplementary references

1. Saraste, J. Spatial and Functional Aspects of ER-Golgi Rabs and Tethers. *Frontiers in Cell and Developmental Biology* **2016**, *4*, doi:10.3389/fcell.2016.00028.
2. Turner, D.L.; Korneev, D. v.; Purdy, J.G.; de Marco, A.; Mathias, R.A. The Host Exosome Pathway Underpins Biogenesis of the Human Cytomegalovirus Virion. *Elife* **2020**, *9*, 1–29, doi:10.7554/ELIFE.58288.
3. Couté, Y.; Kraut, A.; Zimmermann, C.; Büscher, N.; Hesse, A.M.; Bruley, C.; de Andrea, M.; Wangen, C.; Hahn, F.; Marschall, M.; et al. Mass Spectrometry-Based Characterization of the Virion Proteome, Phosphoproteome, and Associated Kinase Activity of Human Cytomegalovirus. *Microorganisms* **2020**, *8*, 1–20, doi:10.3390/microorganisms8060820.
4. Rieder, F.J.J.; Kastner, M.T.; Hartl, M.; Puchinger, M.G.; Schneider, M.; Majdic, O.; Britt, W.J.; Djinović-Carugo, K.; Steininger, C. Human Cytomegalovirus Phosphoproteins Are Hypophosphorylated and Intrinsically Disordered. *Journal of General Virology* **2017**, *98*, 471–485, doi:10.1099/jgv.0.000675.
5. Reyda, S.; Büscher, N.; Tenzer, S.; Plachter, B. Proteomic Analyses of Human Cytomegalovirus Strain AD169 Derivatives Reveal Highly Conserved Patterns of Viral and Cellular Proteins in Infected Fibroblasts. *Viruses* **2014**, *6*, 172–188, doi:10.3390/v6010172.
6. Flomm, F.J.; Soh, T.K.; Schneider, C.; Britt, H.M.; Thalassinos, K.; Pfitzner, S.; Reimer, R.; Grünewald, K.; Bosse, J.B. Egress of Human Cytomegalovirus through Multivesicular Bodies. *bioRxiv* **2021**, 2020.12.31.424954, doi:10.1101/2020.12.31.424954.
7. Birzer, A.; Kraner, M.E.; Heilingloh, C.S.; Mühl-Zürbes, P.; Hofmann, J.; Steinkasserer, A.; Popella, L. Mass Spectrometric Characterization of HSV-1 L-Particles From Human Dendritic Cells and BHK21 Cells and Analysis of Their Functional Role. *Frontiers in Microbiology* **2020**, *11*, doi:10.3389/fmicb.2020.01997.
8. Jeppesen, D.K.; Fenix, A.M.; Franklin, J.L.; Higginbotham, J.N.; Zhang, Q.; Zimmerman, L.J.; Liebler, D.C.; Ping, J.; Liu, Q.; Evans, R.; et al. Reassessment of Exosome Composition. *Cell* **2019**, *177*, 428-445.e18, doi:10.1016/j.cell.2019.02.029.
9. Müller, M.P.; Goody, R.S. Molecular Control of Rab Activity by GEFs, GAPs and GDI. *Small GTPases* **2018**, *9*, 5–21, doi:10.1080/21541248.2016.1276999.
10. Wang, T.; Li, L.; Hong, W. SNARE Proteins in Membrane Trafficking. *Traffic* **2017**, *18*, 767–775, doi:10.1111/tra.12524.
11. McNally, K.E.; Cullen, P.J. Endosomal Retrieval of Cargo: Retromer Is Not Alone. *Trends in Cell Biology* **2018**, *28*, 807–822, doi:10.1016/j.tcb.2018.06.005.
12. Chen, K.E.; Healy, M.D.; Collins, B.M. Towards a Molecular Understanding of Endosomal Trafficking by Retromer and Retriever. *Traffic* 2019, *20*, 465–478.
13. Solinger, J.A.; Rashid, H.O.; Prescianotto-Baschong, C.; Spang, A. FERARI Is Required for Rab11-Dependent Endocytic Recycling. *Nature Cell Biology* **2020**, *22*, 213–224, doi:10.1038/s41556-019-0456-5.
14. Gillingham, A.K.; Munro, S. Transport Carrier Tethering – How Vesicles Are Captured by Organelles. *Current Opinion in Cell Biology* **2019**, *59*, 140–146, doi:10.1016/j.ceb.2019.04.010.
15. Koumandou, V.L.; Dacks, J.B.; Coulson, R.M.R.; Field, M.C. Control Systems for Membrane Fusion in the Ancestral Eukaryote; Evolution of Tethering Complexes and SM Proteins. *BMC Evol Biol* **2007**, *7*, doi:10.1186/1471-2148-7-29.
16. Spang, A. Membrane Tethering Complexes in the Endosomal System. *Frontiers in Cell and Developmental Biology* **2016**, *4*, 1–7, doi:10.3389/fcell.2016.00035.
17. Wang, Y.; Huang, S. Golgi Structure Formation, Function, and Post-Translational Modifications in Mammalian Cells. *F1000Res* **2017**, *6*, 1–13, doi:10.12688/f1000research.11900.1.
18. Burke, J.E. Structural Basis for Regulation of Phosphoinositide Kinases and Their Involvement in Human Disease. *Mol Cell* **2018**, *71*, 653–673, doi:10.1016/J.MOLCEL.2018.08.005.
19. Hsu, F.S.; Mao, Y. The Structure of Phosphoinositide Phosphatases: Insights into Substrate Specificity and Catalysis. *Biochim Biophys Acta* **2015**, *1851*, 698, doi:10.1016/J.BBALIP.2014.09.015.
20. Dikic, I.; Elazar, Z. Mechanism and Medical Implications of Mammalian Autophagy. *Nature Reviews Molecular Cell Biology* **2018**, *19*, 349–364, doi:10.1038/s41580-018-0003-4.
21. New, J.; Thomas, S.M. Autophagy-Dependent Secretion: Mechanism, Factors Secreted, and Disease Implications. *Autophagy* 2019, *15*, 1682–1693.
22. Li, L.; Tong, M.; Fu, Y.; Chen, F.; Zhang, S.; Chen, H.; Ma, X.; Li, D.; Liu, X.; Zhong, Q. Lipids and Membrane-Associated Proteins in Autophagy. *Protein and Cell* 2021, *12*, 520–544.
23. Schöneberg, J.; Lee, I.H.; Iwasa, J.H.; Hurley, J.H. Reverse-Topology Membrane Scission by the ESCRT Proteins. *Nature Reviews Molecular Cell Biology* **2016**, *18*, 5–17, doi:10.1038/nrm.2016.121.
